# Young male patients are at elevated risk of developing serious central nervous system complications during acute Puumala hantavirus infection

**DOI:** 10.1186/1471-2334-11-217

**Published:** 2011-08-14

**Authors:** Timo Hautala, Nina Hautala, Saara-Mari Mähönen, Tarja Sironen, Eija Pääkkö, Ari Karttunen, Pasi I Salmela, Olli Vainio, Seppo Rytky, Alexander Plyusnin, Antti Vaheri, Olli Vapalahti, Heikki Kauma

**Affiliations:** 1 Department of Internal Medicine, Oulu University Hospital, Oulu, Finland; 2 Department of Ophthalmology, Oulu University Hospital, Oulu, Finland; 3 Institute of Diagnostics, Department of Medical Microbiology and Immunology, University of Oulu, Oulu, Finland; 4 Department of Diagnostic Radiology, Oulu University Hospital, Oulu, Finland; 5 Department of Clinical Neurophysiology, Oulu University Hospital, Oulu, Finland; 6 Department of Virology, Infection Biology Research Program, Haartman Institute, University of Helsinki, Helsinki, Finland; 7 Clinical Microbiology Laboratory, Oulu University Hospital, Oulu, Finland

**Keywords:** hantavirus, encephalitis, hypopituitarism

## Abstract

**Background:**

Our aim was to characterize clinical properties and laboratory parameters in patients with or without cerebrospinal fluid (CSF) findings suggestive of central nervous system (CNS) involvement, and especially those who developed serious CNS complications during acute nephropathia epidemica (NE) caused by Puumala hantavirus (PUUV) infection.

**Methods:**

A prospective cohort of 40 patients with acute NE and no signs of major CNS complications was analyzed. In addition, 8 patients with major CNS complications associated with NE were characterized. We collected data of CNS symptoms, CSF analysis, brain magnetic resonance imaging (MRI) results, electroencephalography (EEG) recordings, kidney function, and a number of laboratory parameters. Selected patients were evaluated by an ophthalmologist.

**Results:**

Patients with a positive CSF PUUV IgM finding or major CNS complications were more often males (p < 0.05) and they had higher plasma creatinine values (p < 0.001) compared to those with negative CSF PUUV IgM. The degree of tissue edema did not explain the CSF findings. Patients with major CNS complications were younger than those with negative CSF PUUV IgM finding (52.9 vs. 38.5 years, p < 0.05). Some patients developed permanent neurological and ophthalmological impairments.

**Conclusions:**

CNS and ocular involvement during and after acute NE can cause permanent damage and these symptoms seem to be attributable to true infection of the CNS rather than increased tissue permeability. The possibility of this condition should be borne in mind especially in young male patients.

## Background

Puumala hantavirus (PUUV) causes a hemorrhagic fever with renal syndrome (HFRS) also called Nephropathia epidemica (NE). Although the condition is endemic in Northern Europe, an increasing number of cases has been reported throughout Europe, possibly due to climate change [1-3]. The virus is carried by the bank vole (*Myodes glareolus*); human infection is acquired by inhalation of the virus from aerosolized excreta [1]. After an incubation period of two to four weeks, the patient develops symptoms, i.e. fever, headache, abdominal pain, nausea, diarrhoea, renal insufficiency, and acute myopia [1].

Many patients with acute NE display signs of central nervous system (CNS) involvement [4-7] and evidence of PUUV infection in the CNS has been provided [8,9]. Pituitary hemorrhage as a potential complication of NE has been documented [7,8]. Headache, insomnia, vertigo, nausea, nuchal rigidity, confusion, generalized seizures, acute disseminated encephalomyelitis have been reported and patients with meningoencephalitis after exposure to PUUV have been described [5,6,10-13]. In general, however, hantaviruses may not have been recognized as a typical causative agent of viral encephalitis.

In this study, we characterized the clinical properties of patients with encephalitis or significant CNS injury caused by acute PUUV infection. We also compared the clinical properties of NE patients with positive CSF PUUV IgM finding to those negative for CSF PUUV IgM. Our intention was to identify patient populations that might be at elevated risk of developing significant CNS illness associated with acute NE. In addition, our aim was to analyze the clinical findings in order to search for an explanation for the CNS involvement.

## Methods

### Study design

The patients of this study were partly recruited from those hospitalized for acute NE in the Oulu University Hospital in Northern Finland during September 2005 to February 2008 and the patient population in full has been described in detail elsewhere [7]. For the current study, the data were collected from the 40 patients without major CNS complications and from whom cerebrospinal fluid (CSF) PUUV IgM analysis was available. In addition, 8 patients who had been treated and identified to have suffered major CNS complications caused by acute NE at Oulu University Hospital were included to the study. Their hospital records and clinical findings were scrutinized. These patients included three previously published cases (patients 6-8 in Tables 1 and 2) who suffered severe CNS damage caused by PUUV [8]. The follow-up of each patient was organized individually based on their symptoms.

**Table 1:** Selected laboratory findings of the 8 patients experiencing major CNS complications

Patient	CSF-PUUV IgM	CSF-white cells	CSF-protein	thrombo-cytes	B-white cells	B-Hb	P-CRP	P-Creatinine
1	+	5	448	62	9,9	152	46	609

2	+	0	3265	8	20,5	184	113	353

3	na	na	na	28	13,3	229	115	232

4	+	5	1155	21	15,1	192	223	411

5	na	9	715	13	23,2	201	248	460

6	na	na	na	67	6,9	149	na	295

7	na	18	1526	8	13,8	191	131	505

8	na	2	706	11	3,9	139	169	600

The CSF PUUV IgM was determined in three patients. CSF white cell count (x10e6/l) and protein concentration (mg/l) are shown. The nadir blood thrombocyte count (x10e9/l), blood white cell count (x10e9/l), and haemoglobin concentration (g/l) at the time of hospital entry are shown. The peak plasma CRP (mg/l) and creatinine (μmol/l) concentrations are also provided in the table.

**Table 2:** Summary of the clinical properties of the 8 patients with major CNS involvement caused by acute NE

Patient	gender	age	year	clinical symptoms	MRI finding	hormonal status	EEG
1	male	17	2009	confusion, seizures	cortical oedema	normal	excessive sleepiness

2	male	47	2006	confusion, somnolence	normal	normal	na

3	male	33	2006	transient vision loss, vomiting	pituitary hemorrhage	panhypopuitarism, recovered	normal

4	male	49	2007	confusion, somnolence, transient vision loss	pituitary hemorrhage	panhypopuitarism, hormonal replacement	normal

5	female	47	2005	confusion, somnolence	pituitary hemorrhage	panhypopuitarism, hormonal replacement	mildly increased delta activity

6	male	58	1999	transient vision loss, sudden death	na	pituitary hemorrhage in autopsy	na

7	male	38	1991	confusion, somnolence	pituitary hemorrhage	panhypopuitarism, hormonal replacement	mild disturbance

8	male	19	2000	confusion, seizures	pituitary hemorrhage	panhypopuitarism, recovered	diffuse delta activity and temporal periodic postictal slowing

Gender, age, year of their acute NE, brief description of their illness, brain MRI, hormonal status, and EEG is given.

We compared the clinical presentation of acute NE without major CNS complications between the patients with CSF PUUV IgM-negative cases (21 patients) to those with CSF PUUV IgM-positive findings (19 patients) as well as with the patients who suffered major CNS complications (8 patients). The study protocol was explained to each patient and they had the right to refuse or withdraw from the study according to the Helsinki declaration. The study was approved by the Ethics Committee of the Oulu University Hospital and all participants signed an informed consent form.

### Definition of major CNS complications

Eight patients were identified to have experienced major CNS complications associated with acute NE (Table 2). These patients suffered either pituitary haemorrhage in their brain MRI and subsequent hormonal insufficiency or they exhibited symptoms of significant clinical encephalitis based on the criteria of Centers for Disease Control and Prevention (CDC)[14].

### Clinical data

Patients were examined as previously described in detail [7]. Briefly, CNS symptoms were documented, cerebrospinal fluid (CSF) samples were collected, brain magnetic resonance imaging (MRI) was acquired, and electroencephalography (EEG) was recorded as previously described [7]. In order to estimate the degree of tissue oedema and the disturbance of tissue permeability, the weight change during hospitalization, urine output during the first day of treatment, as well as the plasma creatinine level were recorded. Data of blood haemoglobin (Hb), thrombocyte count, white blood cell count, serum albumin concentration, plasma C-reactive protein (CRP) concentration, CSF protein and albumin concentrations, and CSF white cell count were also collected. Selected patients were examined by an ophthalmologist as described earlier [15]. Patients with suspicion of pituitary injury in their MRI were evaluted for signs of hormonal deficiencies as described previously [7].

### Virological and immunological analysis

The laboratory diagnosis of NE was based on PUUV serology that was initially analyzed using a commercial enzyme-linked immunosorbent assay of IgM antibodies (Reagena Puumala IgM EIA Kit, Reagena, Toivala, Finland). In some cases, the samples were also analyzed with an indirect immunofluorescence test for PUUV-IgG which displayed a granular staining pattern in cases of typical acute infection [16]. The CSF samples were analyzed for cell count, glucose and protein concentrations and CSF and serum PUUV IgM and IgG antibody levels were titrated as described [7]. As previously described, the CSF sample of only one patient was positive for PUUV RNA [9].

### Data analysis

The patients were divided into three categories: those without major CNS complications and negative CSF PUUV-IgM (21 patients), patients without CNS complications and a positive for CSF PUUV-IgM finding (19 patients), and those with major CNS complications caused by acute NE (8 patients). In addition, we explored the clinical data of the patients positive for CSF PUUV-IgM and elevated CSF white cell count (data not shown in the tables). The results were analyzed with Chi-square test, Pearson bivariate correlation test, or t-test (PASW version 18, SPSS Inc., Chicago, IL, USA) for statistical significance.

## Results

### Patients without major CNS complications

#### CNS-related symptoms without major CNS complications

Our first task was to analyze if the laboratory findings differed between the patients with or without symptoms associated with affected CNS during acute NE. We found that all 21 patients with negative CSF PUUV IgM as well as nearly all patients (18/19, 95%) with positive CSF PUUV IgM complained of symptoms suggestive of CNS involvement (headache, nausea, vertigo). Since such a high proportion of the patients did experience CNS-related symptoms, no statistically significant differences in age, gender, any CSF finding, or other clinical or laboratory parameters based on these symptoms could be detected.

#### CSF PUUV IgM

The CSF PUUV IgM-positive finding may be considered to indicate intrathecal antibody production and the possibility of a true CNS infection. Based on this hypothesis, we compared the properties of IgM-positive and -negative patients. Selected clinical and laboratory findings of the study patients without major CNS complications divided according to CSF PUUV IgM-negative or -positive finding are summarized in Table 1. When compared to those who were negative for the PUUV IgM the patients who were positive for CSF PUUV IgM were more often male (p < 0.05, chi-square test), their mean plasma creatinine concentration was higher (p < 0.001, t-test), the CSF protein value elevated (p < 0.001, t-test), and their blood white cell count was high (p < 0.001, t-test). No correlation was seen between the CSF PUUV IgM and urine output, weight change during hospitalization, or blood pressure values. In addition, the severity of thrombocytopenia or the plasma CRP concentration was not associated with the positive CSF PUUV IgM finding. Similar results were seen in the subcategory of patients in whom both the CSF PUUV IgM was positive and the CSF white cell count was elevated. Time from the onset of NE symptoms to lumbar puncture did not differ between the CSF PUUV IgM positive and negative patients.

#### CSF protein concentration and white cell count

It is possible that the CSF protein concentration and white cell count can be elevated either due to a true PUUV infection in the CNS or because of increased tissue permeability caused by the acute NE. We explored the possibility that clinical and laboratory parameters should possibly explain the mechanisms of the CSF findings. There was a statistically significant correlation between the CSF protein concentration and the severity of thrombocytopenia (p < 0.05, Pearson bivariate correlation test). The patients with elevated CSF protein concentration had a high blood white cell count (p < 0.05, Pearson bivariate correlation test) and there was a correlation between the CSF protein concentration and the plasma creatinine level (p < 0.05, Pearson bivariate correlation test). In contrast, the degree of tissue edema or urine output displayed no association with the CSF protein concentration or white cell count. We were able to analyze albumin concentrations from 26 CSF samples in order to investigate possible blood-brain barrier injury. We found that CSF albumin concentration was higher in patients with positive PUUV IgM CSF finding compared to PUUV IgM negative cases (p < 0.01, t-test). The CSF/serum albumin ratio, however, was not different between the CSF PUUV IgM positive and negative cases. In addition, the CSF albumin concentrations were similar in the CSF samples with elevated white cell count compared to those with low CSF white cell count.

#### Kidney failure and tissue edema

The patients with high plasma creatinine levels also exhibited the most significant tissue edema as demonstrated by the correlation between with the weight change during hospitalization and plasma creatinine concentration (p < 0.05, Pearson bivariate correlation test). These patients also had the lowest urine output during the first day of treatment (p = 0.01, Pearson bivariate correlation test). As mentioned above, the patients with high plasma creatinine concentration had high CSF protein concentrations (p < 0.05, Pearson bivariate correlation test) and they also had a high blood white cell count (p < 0.05, Pearson bivariate correlation test).

### Patients with major CNS involvement

#### CNS symptoms

Selected clinical, imaging, and laboratory findings of patients with major CNS involvement are summarized in Tables 1, 2, and 3. All patients experienced compromised alertness, confusion, and somnolence at some point during the course of their illness and two patients developed seizures. The five patients that are summarized in Table 2 developed pituitary insufficiency; two of them recovered spontaneously but three patients continued to require hormonal replacement therapy. One patient developed (patient 4 in Table 1 and 2) a significant neurological condition: this male patient was healthy and free of medication before his acute NE in 2007. During the acute illness, he suffered nausea, significant pulmonary edema, somnolence, and impaired awareness and he had periods of complete loss of vision. He developed permanent pituitary insufficiency and also, he developed a prolonged cognitive impairment and severe neuralgia on the left side of his head. In addition, he developed weakness of his left limbs. It appears that his neurological condition is irreversible and this patient has been unable to return to work. Repeated MRI of the brain and angiography of his head and neck have not revealed any vascular reason for his neurological symptoms.

**Table 3:** The table summarizes the clinical and laboratory parameters studied in the NE patients categorized according to their major CNS complications and CSF PUUV IgM findings

	No major CNS complications (n = 21)	No major CNS complications (n = 19)	Patients with major CNS complications (n = 8)
CSF PUUV IgM	negative	positive	

Female (%)	47.6	15.8*	12.5*

Age (years)	52.9	45.7	38.5*

CSF white cells (x10e6/l)	1.5	4.7	6.5**

CSF protein (mg/l)	387	780**	1302***

B-thrombocytes (x10e9/l)	80	62	27**

B-white cells (x10e9/l)	7.4	11.3**	13.3**

B-Hb (g/l)	149	152	179**

P-CRP (mg/l)	100	63	149*

P-Creatinine (umol/l)	113	413***	433***

BP, systolic (mmHg)	129	129	143

hospitalization (days)	5.0	5.1	9.3***

dialysis	0	0	0

In each category, the percentage of female gender, mean age of the patients, mean CSF white cell count, and mean CSF protein concentration are given. Blood thrombocyte count (nadir), peak plasma creatinine level, blood white cell count and haemoglobin concentration at the time of hospital entry are reported. Systolic blood pressure (BP) at the time of hospital entry, length of hospitalization, and number of patients treated with dialysis are given. Statistical analysis is a comparion between the CSF PUUV IgM negative and positive cases, and the CSF PUUV IgM negative cases and the patients with major CNS complications. * indicates p < 0.05, ** p < 0.01, and *** p < 0.001.

#### MRI imaging

The imaging findings of the patients with major CNS complications caused by NE are summarized in Table 2. Five patients had pituitary hemorrhage in their MRI and they all developed panhypopituitarism. Three patients (patients 2, 5, and 7, Table 2) experienced symptoms typical of clinical encephalitis although their brain MRI showed hemorrhage only in the pituitary.

#### Ophthalmological symptoms and findings in patients with severe CNS involvement

Three male patients with major CNS findings experienced a sudden, transient, and complete loss of vision for less than 5 minutes associated with severe headache, vomiting, and dizziness immediately before or during their hospitalization (patients 3, 4, and 6). One patient (patient 2) experienced a significant reduction of visual acuity to hand movement level, light sensitivity, and ocular pain during the first days of hospitalization. Another patient (patient 4) suffered from intermittent blurring of vision, diplopia, sense of defects in visual field, and nystagmus for at least 18 months after the acute NE. His visual field appeared normal in both Goldmann and Humphrey 24-2 perimetry and no abnormalities were noted in diplopia test.

A tendency towards myopia in the acute phase compared to control examination was noted in the three patients (patients 2 - 4 in Table 2) with major CNS findings (mean refraction change 0.7 D between acute and control examination). A major decline in intraocular pressure (IOP; mean change 6.8 mmHg, from 15.0 mmHg to 8.1 mmHg) was measured in the two patients (patients 3 and 4) with a pituitary injury in the acute phase of NE compared to the control examination. Interestingly, in one of these patients (patient 4) with the pituitary injury, the IOP has remained at a significantly lower level compared to that documented during his previous ophthalmic evaluations even after the clinical recovery. No change in anterior chamber depth was noted in these patients. Compared to the control examination, the acute-phase mean 0.9 mm deepening of the crystalline lens was noted in the three patients with major CNS findings (from mean 5.0 mm to mean 4.1 mm). In the acute phase compared to control examination, the length of the vitreous cavity was shallower in the patients with CNS findings (mean difference 0.5 mm, p < 0.01) than in the other patients with acute NE (mean difference 0.2 mm). In addition, axial length of the eye was greater in patients with CNS findings in the acute phase compared to the control examination (mean difference 0.35 mm).

## Discussion

Our patient population and previous case reports strongly suggests that acute NE may indeed cause severe CNS complications [5-8,12,13]. The clinical presentation of many of our patients included typical symptoms of viral encephalitis and several patients developed serious CNS-related complications. It seems understandable that PUUV hantavirus should be considered as a possible causative viral agent in patients with encephalitis, at least in endemic regions. Due to the expanding nature of the NE epidemic and increasing international tourism, the possibility of this clinical condition should also be borne in mind elsewhere. It should be appreciated, however, that the patient population of this study represents the most severely affected cases; majority of NE patients do not require hospitalization and many of them should recover without complications.

The presence of PUUV in the CNS during the acute infection has been demonstrated [8,9]. It is possible that the virus is present in the CNS early during the course of the illness only which may explain the low number of PUUV RNA-positive CSF findings in the previous studies [4,7]. The positive CSF PUUV IgM may be considered to indicate intrathecal antibody production and the finding may be a reflection of a true CNS infection [7]. The severe cases in this current report demonstrate that the acute NE may truly damage the brain and the CNS-related symptoms during the acute NE should not simply be attributable to a transient increase in the tissue permeability. This speculation is supported by the finding that the degree of tissue edema, amount of urine output, or blood pressure level displayed no association with the PUUV IgM-positive CSF findings.

It is generally believed that most patients recover from acute NE without suffering any long-term consequences [1]. Our current report, however, suggests that some patients may actually experience long-lasting CNS impairment. One patient in our current study developed a disabling neurological condition and permanent hormonal insufficiency following acute NE. In addition, he suffered from intermittent blurring of vision, diplopia, a sense of defects in visual field, and nystagmus for at least 18 months after the acute NE. His visual field appeared normal in both Goldmann and Humphrey 24-2 perimetry and no abnormalities were noted in a diplopia test. In addition, the IOP level of this patient remained at a low level even after clinical recovery compared to the value documented in his previous ophthalmic evaluations before the infection. We believe that this may be a consequence of a permanent injury to the ciliary body which occurred during the inflammatory reaction caused by the acute NE. Our observations of these permanent injuries are in agreement with previous studies; these studies concluded that pituitary insufficiency after an acute hantavirus infection may be more common than formerly believed [17,18]. Mechanism of the injury, however, remains unclear. It has been speculated that the pituitary gland is highly vulnerable because of its anatomical location and organization of blood flow [8]. Possibility of thrombosis or vasculitis, however, can not be ruled out. Furthermore, our study failed to produce conclusive data on mechanisms and severity of possible blood-brain barrier injury. Future studies should be designed to answer these open questions.

Our study shows that the CNS involvement during acute NE is dependent on the age and the gender of the patient. Most patients with a serious CNS injury or intrathecal PUUV antibody production were male and, in addition, the patients with major events were younger than those exhibiting no signs of CNS involvement. Our previous study showed lower number of abnormal CSF findings in the patients positive for DR15(2)-DQ6 haplotype [7]. In that population, 86% of the patients with the protective haplotype were male and their mean age was 48 years. These results indicate that our current observation of the young age and the male gender in adult population is not attributable to this protective HLA haplotype. It must be recognized, however, that serious complications caused by NE are rarely seen in children [19,20]. It is possible that testosterone levels may contribute to the severity of the hantavirus infection [21].

Our current report demonstrates that the brain MRI and hormonal studies should be considered at least whenever severe NE is diagnosed in a young male patient. It should be appreciated that the illness may lead to long term neurological and ophthalmological impairments which may be caused by a true CNS infection. At present, little is known about the biological mechanisms leading to injury and further studies should be conducted.

## Conclusion

Acute PUUV hantavirus infection can cause permanent CNS and ocular damage. In our patient population the CNS injury was observed in young adult male patients. It seems possible that these symptoms are attributable to true infection of the CNS rather than increased tissue permeability.

## Competing interests

The authors declare that they have no competing interests.

## Authors' contributions

TH, NH, and HK examined the patients. SMM, TS, OV, AP, AV, and OV have participated to the laboratory analysis. EP and AK analyzed the brain MRI studies. PS is responsible for the endocrinological evaluation. SR analyzed the EEG examinations. All authors have participated to the preparation of the manuscript. All authors read and approved the final manuscript.

## Pre-publication history

The pre-publication history for this paper can be accessed here:

http://www.biomedcentral.com/1471-2334/11/217/prepub
